# Efficacy of a Brief Cognitive Behavioral Therapy for Head and Neck Cancer Survivors with Body Image Distress: Secondary Outcomes from the BRIGHT Pilot Randomized Clinical Trial

**DOI:** 10.21203/rs.3.rs-3222601/v1

**Published:** 2023-08-07

**Authors:** Evan M. Graboyes, Emily Kistner-Griffin, Elizabeth G. Hill, Stacey Maurer, Wendy Balliet, Amy M. Williams, Lynne Padgett, Flora Yan, Angie Rush, Brad Johnson, Taylor McLeod, Jennifer Dahne, Kenneth J. Ruggiero, Katherine R. Sterba

**Affiliations:** Medical University of South Carolina; Medical University of South Carolina; Medical University of South Carolina; Medical University of South Carolina; Medical University of South Carolina; Corewell Health; United States Department of Veterans Affairs; Temple University; Head and Neck Cancer Alliance; Head and Neck Cancer Alliance; Medical University of South Carolina; Hollings Cancer Center, MUSC; Medical University of South Carolina; Medical University of South Carolina

**Keywords:** survivorship, body image, head and neck cancer, cognitive behavioral therapy, telemedicine, psychosocial oncology

## Abstract

**Purpose:**

Body image distress (BID) among head and neck cancer (HNC) survivors leads to depression, social isolation, stigma, and poor quality of life. BRIGHT (Building a Renewed ImaGe after Head & neck cancer Treatment) is a brief, tailored cognitive behavioral therapy (CBT) that reduces HNC-related BID. This trial examines the effect of BRIGHT on psychosocial outcomes among HNC survivors with BID.

**Methods:**

In this pilot randomized trial, HNC survivors with clinically significant BID were randomized to 5 weekly psychologist-led tele-CBT sessions (BRIGHT) or dose-and delivery matched survivorship education (attention control [AC]). Secondary psychosocial outcomes were assessed using validated patient-reported outcomes at baseline and 1- and 3-months post-intervention.

**Results:**

Among 44 HNC survivors with BID, BRIGHT resulted in a greater reduction in depression relative to AC (mean model-based 1-month difference in Δ PROMIS SF v1.0-Depression 8a score, −3.4; 90% CI, −6.4 to −0.4; 3-month difference, −4.3; 90% CI, −7.8 to −0.8). BRIGHT also decreased shame and stigma relative to AC (mean model-based 3-month difference in Δ Shame and Stigma Scale score, −9.7; 90% CI, −15.2 to −4.2) and social isolation (mean model-based 3-month difference in Δ PROMIS SF v2.0 Social Isolation 8a score, −2.9; 90% CI, −5.8 to −0.1).

**Conclusions:**

In this planned secondary analysis of a pilot RCT, BRIGHT improved a broad array of psychosocial outcomes among HNC survivors with BID.

**Implications for Cancer Survivors:**

These promising preliminary data suggest the need for a large efficacy trial evaluating the effect of BRIGHT on psychosocial outcomes among HNC survivors with BID.

**Trial Registration:**

ClinicalTrials.gov identifier: NCT03831100

## Introduction

Head and neck cancer (HNC) and its treatment result in substantial life-altering changes related to facial disfigurement, difficulty swallowing, impaired smiling, and challenges speaking.^1^ Because these changes are often highly visible and affect daily social function, 75–90% of HNC survivors express body image concerns^2,3^ and up to 28% have clinically significant body image-related distress (BID).^4,5^ BID results in devastating psychosocial morbidity and functional impairment.^6^ In fact, HNC survivors with BID experience a 6-fold increase in moderate-severe depressive symptoms, an 8-fold increase in moderate-severe anxiety symptoms, and increased rates of social isolation and feelings of stigmatization.^4,7-13^ As a result, BID among HNC survivors is associated with reduced quality of life (QOL) and may contribute to the two-fold higher rate of suicide mortality relative to other cancer survivors.^4,14^

Although BID is a critical survivorship issue for patients with HNC,^15^ evidence-based strategies to manage BID among HNC survivors are lacking.^16^ However, cognitive behavioral therapy (CBT)-based approaches are emerging as potential treatment paradigms for these patients.^17,18^ Our team developed BRIGHT (Building a Renewed ImaGe after Head & neck cancer Treatment) as a brief tailored CBT that targets maladaptive body image coping skills among HNC survivors.^19^ Our previous single-arm trial demonstrated that BRIGHT was both feasible and acceptable.^19^ Additionally, it improved BID and HNC-related QOL at 1- and 3-month post-intervention.^19^ In a subsequent pilot randomized clinical trial (RCT), we showed that BRIGHT decreased cancer-related BID and HNC-related BID at 1- and 3-months post-intervention relative to dose and delivery-matched attention control (AC).^20^ However, the effect of BRIGHT on associated psychosocial outcomes (e.g., depression, anxiety, shame and stigma, social isolation) among HNC survivors with BID is not known. In this analysis, we examined the effect of BRIGHT on a broad range of pre-defined secondary psychosocial outcomes among HNC survivors with BID.

## Methods

### Study Approval and Guidelines

The study and protocol were approved by the institutional review board at the Medical University of South Carolina (MUSC). The CONSORT diagram (**eFigure 1** in **Supplement 1**), methods, and primary analyses for the pilot RCT have been described in detail.^20^ Results are reported according to the Consolidated Standards of Reporting Trials Extension (CONSORT Extension) reporting guidelines for randomized pilot and psychological intervention trials.^21,22^

### Patients and Study Procedures

Patients were recruited from the MUSC HNC clinic during a routine survivorship encounter. Eligible patients were adult HNC survivors with clinically significant BID, as determined by a Body Image Scale score ≥ 10.^23^ Following written informed consent and completion of baseline assessments, patients were randomized 1:1 to BRIGHT or AC using a permuted block randomization design with block sizes of 4 or 6. Study outcomes were assessed at baseline, 1-month post-intervention, and 3-months post-intervention.

### Interventions

In brief, BRIGHT is a theory-based,^24-28^ manualized CBT consisting of 5 weekly 60-minute sessions delivered one-on-one by a licensed clinical psychologist via a video telemedicine platform. BRIGHT session topics include (1) psychoeducation about the cognitive model of body image; (2) self-monitoring about thoughts, feelings, and body image behaviors; (3) cognitive restructuring to identify and challenge unhelpful automatic HNC-related body image thoughts; (4) adaptive body image coping strategies; and (5) maintenance and relapse prevention. AC is a tele-supportive care intervention consisting of educational videos that address non-body image aspects of HNC survivorship (e.g., financial toxicity, fear of cancer recurrence, etc). Following best practices for choosing control groups within behavior change RCTs,^29^ we designed AC to match BRIGHT’s dose (5 weekly sessions) and delivery method (video-based telemedicine) while not providing the behavior change mechanism in BRIGHT. Fidelity and adherence to both arms have previously been described.^20^ AC was not delivered by a mental health professional; adherence to AC was monitored by a member of the research team.

### Study Measures

Demographic data were collected via self-report; clinical characteristics were extracted from the electronic health record. Patient-reported outcomes were administered electronically using REDCap. The primary analyses for acceptability, cancer-related BID (as measured by the Body Image Scale^30^) and HNC-related BID (as measured by the IMAGE-HN^31^) have been previously reported.^20^

Secondary outcomes related to psychosocial morbidity are described below. Depression was assessed with the PROMIS SF v1.0-Depression 8a, a validated, 8-item measure developed by the NIH to assess patient-reported negative mood, views of self, and decreased positive affect and engagement.^32^ Anxiety was measured by the PROMIS SF v1.0-Anxiety 8a, a validated, 8-item measure developed by the NIH to assess patient-reported fear, worry, and hyperarousal.^32^ Satisfaction with social roles was measured by the PROMIS SF v2.0-Satisfaction with Social Roles and Activities 8a, a validated, 8-item, measure developed by the NIH to assess patient-reported satisfaction with performing one’s usual social roles and activities.^33^ Social isolation was assessed with the PROMIS SF v2.0-Social Isolation 8a, a validated, 8-item measure developed by the NIH to assess self-reported perceptions of being avoided, excluded or unknown by others.^33^ For each PROMIS measure, the total score ranges from 8 to 40, with higher scores representing more severe symptoms of the construct measured (e.g., depression, anxiety). HNC-related shame and stigma were measured with the Shame and Stigma Scale, a validated 20-item, unidimensional patient-reported outcome measure of shame regarding appearance, stigma, regret, and social/speech concerns in patients with HNC over the prior 7 days.^12^ The total score ranges from 0 to 80, with higher scores representing greater shame and stigma from HNC.

### Statistical Analysis

Data collected from the study population were included in all descriptive and model-based analyses. All analyses were conducted using Rstudio (R version 4.2.3). Means and standard deviations were summarized by treatment group for each of the secondary outcomes of interest (Shame and Stigma Scale, PROMIS SF v1.0-Depression 8a score, PROMIS SF v1.0-Anxiety 8a, PROMIS SF v2.0-Social Isolation 8a and PROMIS SF v2.0-Satisfaction with Social Roles and Activities 8a) at baseline, 1-month post-intervention and 3-months post-intervention. The effect of BRIGHT on the 1-month and 3-month change scores for each outcome was estimated using analysis of covariance. All models of change scores also included the baseline values as covariates to adjust for correlation between the change score and the baseline values. Treatment effects were estimated as a function of treatment group modeled as a 3-level variable (AC, BRIGHT & Therapist A, or BRIGHT & Therapist B) to account for heterogeneity in therapist effect. The overall treatment effect was estimated using a model-based contrast with a weighted average across therapists in the BRIGHT arm (therapist A weight = 13/20 and therapist B weight = 7/20) and contrast equal to (13/20) β_1_ + (7/20) β_2_ as therapist A conducted BRIGHT for 13 subjects and therapist B conducted BRIGHT for 7 subjects. Statistical testing was 2-sided,with P < .10 considered statistically significant; 90% confidence intervals (CIs) were reported for point estimates. Waterfall plots of subject-level change scores were generated as well as plots of unadjusted means and 90% CIs for change scores in BRIGHT and AC treatment arms at 1-month and 3-months post intervention.

## Results

Baseline characteristics are shown in **eTable 1 in Supplement 1**. Patients had a median age of 63 years (range, 41–80 years) and 61% (27/44) identified as female. The most common head and neck subsite was the oral cavity (50%; 22/44); 61% of patients (27/44) had stage III/IV HNC, and 61% (27/44) received adjuvant (chemo)radiation.

### Depression and Anxiety

**Figure 1:**
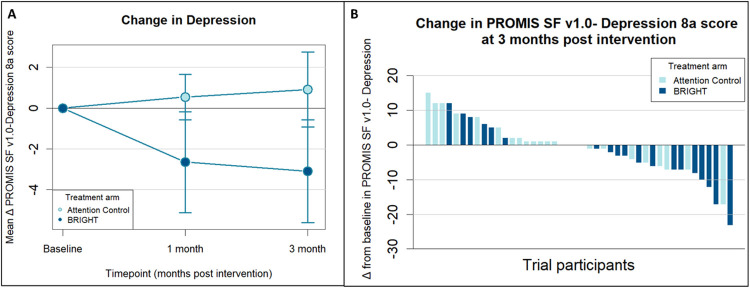
Mean Change from Baseline and Response of Depression for Patients in BRIGHT and Attention Control **A)** Line graph demonstrating the mean change from baseline in PROMIS SF v1.0-Depression 8a scores over time by intervention allocation. Error bars represent 1 SE above and below the mean. **B)** Waterfall plot showing response to BRIGHT (Building a Renewed ImaGe after Head and neck cancer Treatment) and Attention Control as measured by change from baseline in PROMIS SF v1.0-Depression 8a at 3 months post-intervention. The PROMIS SF v1.0-Depression 8a score ranges from 8 to 40, with higher change scores indicating worse depression (and negative bars thus indicating improvement in depression).

BRIGHT decreased depression from baseline to 1-month post-intervention relative to AC (mean model-based difference in change in PROMIS SF v1.0-Depression 8a score, −3.4 points; 90% CI, −6.4 to −0.4 points) and from baseline to 3 months post-intervention relative to AC (mean model-based difference in change in PROMIS SF v1.0-Depression 8a score, −4.3 points; 90% CI, −7.8 to −0.8 points; Table 1). Figure 1 shows the mean change from baseline in PROMIS SF v1.0-Depression 8a as well as the waterfall plot of change in depression from baseline to 3 months post-intervention. BRIGHT did not reduce anxiety at 1- or 3-most post-intervention relative to AC (Table 1).

### HNC-Related Shame and Stigma

**Figure 2:**
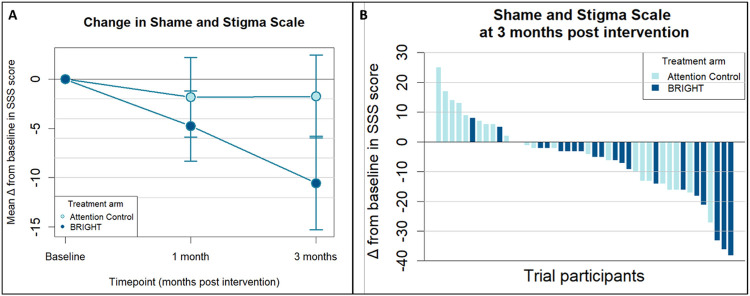
Mean Change from Baseline and Response of Head and Neck Cancer-Related Shame and Stigma for Patients in BRIGHT and Attention Control **A)** Line graph demonstrating the mean change from baseline in Shame and Stigma Scale scores over time by intervention allocation. Error bars represent 90% confidence intervals around the mean changes. **B)** Waterfall plot showing response to BRIGHT (Building a Renewed ImaGe after Head and neck cancer Treatment) and Attention Control as measured by change from baseline in Shame and Stigma Scale at 3 months post-intervention. The Shame and Stigma Scale score ranges from 0 to 80, with higher scores indicating worse HNC-related shame and stigma (and negative bars thus indicating improvement in HNC-related shame and stigma).

Figure 2A shows the mean change from baseline over time in Shame and Stigma Scale scores for patients allocated to BRIGHT and AC. The waterfall plot demonstrating each patient’s clinical response to BRIGHT or AC, as measured by change in Shame and Stigma Scale scores from baseline to 3 months post-intervention, is shown in Fig. 2B. BRIGHT decreased HNC-related shame and stigma at 3-month post-intervention relative to AC (mean model-based difference in Shame and Stigma Scale score, −9.7 points; 90% CI, −15.2 to −4.2 points; Table 1).

### Social Isolation and Satisfaction with Social Activities

**Figure 3:**
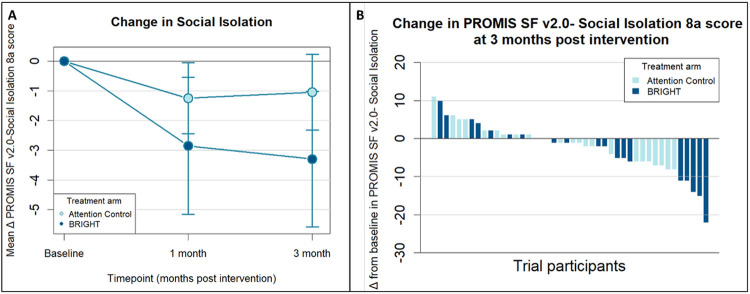
Mean Change from Baseline and Response of Social Isolation for Patients in BRIGHT and Attention Control **A)** Line graph demonstrating the mean change from baseline in PROMIS SF v2.0-Social Isolation 8a scores over time by intervention allocation. Error bars represent 90% confidence intervals around the mean change. **B)** Waterfall plot showing response to BRIGHT (Building a Renewed ImaGe after Head and neck cancer Treatment) and Attention Control as measured by change from baseline in PROMIS SF v2.0-Social Isolation 8a at 3 months post-intervention. The PROMIS SF v1.0-Depression 8a score ranges from 8 to 40, with higher scores indicating worse depression (and negative bars thus indicating improvement in depression).

The effect of BRIGHT on social isolation and satisfaction with social activities at 1- and 3-months post-intervention was mixed (Table 1). While BRIGHT decreased social isolation from baseline to 3-months post-intervention relative to AC (mean model-based difference in change in PROMIS Social Isolation 8a score, −2.9 points; 90% CI, −5.8 to −0.1 points), there was no effect on social isolation at 1-month post-intervention (mean model-based difference in change in PROMIS Social Isolation 8a score, −2.2 points; 90% CI, −5.2 to 0.7 points). The mean change from baseline in social isolation and waterfall plot of change in social isolation from baseline to 3 months post-intervention are shown in Fig. 3. BRIGHT improved satisfaction with social roles and activities at 1-month post-intervention relative to AC (mean model-based difference in change in PROMIS Satisfaction with Social Roles and Activities 8a score, 3.3 points; 90% CI, 0.2 to 6.3 points), but not at 3-months post-intervention (mean model-based difference in change in PROMIS Satisfaction with Social Roles and Activities 8a score, 2.7 points; 90% CI, −0.4 to 5.7 points).

## Discussion

This planned analysis of secondary outcomes of a pilot RCT aimed to elucidate the effect of BRIGHT, a brief tailored CBT, on a broad array of psychosocial outcomes among HNC survivors with BID. Herein we report preliminary data suggesting that BRIGHT reduces depression, shame and stigma and social isolation, and improves satisfaction with social roles and activities among HNC survivors with BID relative to AC. For each psychosocial outcome measure improved by BRIGHT, we also show that the beneficial effects of BRIGHT are realized by most patients and that the responses are relatively durable, with sustained and even increased improvement at 3-months post-intervention. When considered in conjunction with the primary outcome data from this pilot RCT,^20^ the findings of this current study add to the growing evidence base supporting BRIGHT as a novel, evidence-based strategy to manage BID among HNC survivors.

Depression, although common among HNC survivors,^1,15^ is especially prevalent among HNC survivors with BID.^34^ One recent study reported that HNC survivors with clinically significant BID had a 6-fold increase in moderate to severe depressive symptoms relative to HNC survivors without BID.^10^ Among HNC survivors, depression is associated with reduced QOL,^35^ higher rates of suicide,^36^ and worse overall survival.^37^ When considering trials evaluating the effectiveness of interventions targeting BID among HNC survivors, the effect on depression is thus a critically important endpoint. In this pilot trial, BRIGHT resulted in a statistically significant reduction in depressive symptoms at 1- and 3-months post-intervention. The effect of BID-focused interventions on depression in other trials have had mixed results. For example, a self-compassion based intervention did not improve depression among HNC survivors with BID in a single-arm pre-post study,^38^ whereas a skin camouflage program did improve depression relative to control.^39^ In two other recent trials evaluating interventions to reduce BID among HNC survivors, depression was not evaluated as an endpoint.^18,40^ The reasons for the different observed effect of BID-focused interventions on depression amongst these trials is not known but may relate to the efficacy of the intervention on the targeted outcome (BID), with BRIGHT showing the largest improvement in BID in this population. Whatever the explanation, it is clear that (1) future studies should include depression as a secondary endpoint and (2) these preliminary data suggest that BRIGHT, in addition to reducing HNC-related BID, may also improve a key psychosocial outcome in these patients.

When interpreting the findings from the current trial, it is important to consider the unclear causal and temporal relationship between depression, BID, and QOL. CBT is an effective treatment for depression among cancer survivors and one of the recommended 1 st line therapies for cancer survivors with moderate depression.^41,42^ Thus, one possible interpretation of our findings is that BRIGHT caused a reduction in depression primarily, and the observed improvement in BID is causally downstream of the improvement in depression. It is also possible that there is a bidirectional relationship between depression and BID in which reductions in one results in further reductions in the other. Finally, a third potential interpretation of these data is that BRIGHT improves BID primarily and the reduction in depression is downstream. Although the current study design precludes definitively evaluating the temporal relationship between changes in BID and changes in depression following BRIGHT, we believe this third interpretation is the most likely for two reasons. First, the content and psychotherapeutic techniques of the BRIGHT program focus exclusively on domains related to HNC BID-related and are thus unlikely to be sufficiently therapeutically active to see an improvement in depressive symptoms of this magnitude due to direct effects on depression. Second, limited data from two prospective cohort studies suggest that pre-treatment depression is not a risk factor for HNC-related BID, that BID develops temporally prior to depression in patients for whom the conditions co-occur.^13,43^ Additional research is therefore necessary to disentangle the temporal relationship between changes in depression and BID among HNC survivors following BRIGHT.

Shame, stigma, and social isolation are all important, if understudied, psychosocial outcomes for HNC survivors.^12^ Although common among all HNC survivors, shame and stigma-related concerns are especially prevalent among HNC survivors with disfigurement and other visible differences.^6^ Patients with facial disfigurement and BID limit social interaction and participation in social functions.^44,45^ BID among HNC survivors is also associated with unemployment.^10^ Outside the context of psychosocial oncology, shame and stigma have significant effects on social, family, and professional relationships. Although they are not universally included,^18,38,40^ shame, stigma, and social isolation are all key secondary endpoints for trials evaluating interventions to improve BID among HNC survivors. In this pilot trial, we provide preliminary evidence that BRIGHT reduces HNC-related shame and stigma, decreases social isolation, and improves satisfaction with social roles and activities among HNC survivors with BID. Our data align with other studies which have observed similar benefits of BID-focused interventions on outcomes of social avoidance and fear of social interactions.^39^ These preliminary data suggest that managing BID among HNC survivors with BID through interventions such as BRIGHT may thus contribute to improvements in performance in key social domains. As with depression, the inter-relationship and causal dependency of BID with each of these constructs (and with each of these constructs with one another) is complex. However, based on current conceptual models of HNC-related BID, it is likely that the effect of BRIGHT on shame and stigma, social isolation, and social activities is downstream of its effect on HNC-related BID.

### Limitations

As discussed in the publication of the primary outcomes for this pilot RCT, findings should be interpreted within the context of several trial limitations. Consistent with its pilot objective, the trial had a single-site design, small sample size, and short follow-up.^20^ With regard to the limitations of the specific secondary outcomes analyzed herein, the study was not powered for these outcomes, nor was it powered for more complex analyses that examined the relative associations of BID and depression to quality of life. Therefore, these planned secondary analyses should be considered hypothesis-generating and require confirmation in a larger trial. In addition, the clinical significance of the statistically significant improvements in measures of shame and stigma, depression, social isolation, and satisfaction with social roles and activities is not known. Therefore, further psychometric work is necessary to understand clinically meaningful differences in these measures over time and between groups within this patient population. Finally, although the trial was designed prior to the COVID-19 pandemic, it was conducted primarily during the pandemic when masking and social isolation were recommended public health measures. As a result, the external validity of study findings about the effect of BRIGHT on depression, anxiety, social isolation, and satisfaction with social activities outside the context of COVID-19 related masking (which effectively conceals many facial disfigurements associated with HNC treatment) and social avoidance public health measures is unknown and thus requires further study.

## Conclusions

BID among HNC survivors is common and associated with devastating psychosocial morbidity including depression, anxiety, social isolation, and stigma. In this planned secondary analysis of a pilot RCT, BRIGHT improved a broad array of psychosocial outcomes among HNC survivors with BID. The improvements in depression, HNC-related shame stigma, social isolation, satisfaction with social roles and activities, seen with BRIGHT, when considered in conjunction with the previously reported improvement in HNC-related BID, further support conducting a large multisite efficacy trial to establish BRIGHT as the first evidence-based treatment for HNC survivors with BID.

## Figures and Tables

**Table 1:** Effects of Treatment on Psychosocial Outcomes

	AC (N = 24)	BRIGHT (N = 20)		
Instrument	Summary measures Mean ± SD	Summary measures Mean ± SD	Treatment effect* (90% CI)	p- value
PROMIS SF v1.0-Depression 8a				
Baseline	19.2 ± 7.7	18.8 ± 7.6	–	–
1 month	19.8 ± 8.5	16.1 ± 5.1	−3.4 (−6.4, −0.4)	**0.064**
3 months	20.1 ± 8.1	15.7 ± 6.9	−4.3 (−7.8, −0.8)	**0.046**
PROMIS SF v1.0-Anxiety 8a				
Baseline	20.8 ± 6.9	20.0 ± 7.0	–	–
1 month	20.5 ± 9.8	18.4 ± 7.5	−1.5 (−5.2, 2.1)	0.49
3 months	20.6 ± 8.4	17.0 ± 8.3	−3.1 (−6.5, 0.3)	0.14
Shame and Stigma Scale†				
Baseline	35.4 ± 11.3	33.9 ± 12.1	–	–
1 month	33.5 ± 15.3	29.1 ± 9.5	−3.4 (−8.8, 2.0)	0.30
3 months	33.6 ± 14.5	23.3 ± 6.2	−9.7 (−15.2, −4.2)	**0.005**
PROMIS SF v2.0-Social Isolation 8a				
Baseline	20.9 ± 7.4	19.4 ± 7.7	–	–
1 month	19.7 ± 8.2	16.6 ± 5.8	−2.2 (−5.2, 0.7)	0.22
3 months	19.9 ± 8.0	16.1 ± 5.2	−2.9 (−5.8, −0.1)	**0.093**
PROMIS SF v2.0-Satisfaction with Social Roles and Activities ‡				
Baseline	24.3 ± 8.9	21.7 ± 8.8	−	−
1 month	23.3 ± 9.2	24.8 ± 7.0	3.3 (0.2, 6.3)	0.082
3 months	26.1 ± 7.8	27.9 ± 6.3	2.7 (−0.4, 5.7)	0.15

Abbreviations: AC = Attention Control ; BRIGHT = Building a Renewed ImaGe after Head and neck cancer Treatment; PROMIS = Patient-Reported Outcome Measurement Information System; SF = Short form

* Model-based treatment effect estimated using ANACOVA with change score (1 (or 3)-month score – baseline score) modeled as a function of treatment group (AC, BRIGHT & Therapist A, BRIGHT & Therapist B), with adjustment for baseline scores.

† Shame and Stigma Score in HNC: Q11 omitted due to missing data from systematic error in administration of the survey to the first 18 trial participants.

‡ PROMIS Satisfaction with Social Roles and Activities, there was unit nonresponse at baseline for one patient in AC, precluding change score calculation. Therefore, all summary statistics and modeling related to this variable included only 23 AC subjects.

## Data Availability

We will make de-identified data available to users under a data-sharing agreement that provides for: (1) a commitment to using the data only for research purposes and not to identify any individual participant; (2) a commitment to securing the data using appropriate technology; and (3) a commitment to destroying or returning the data after analyses are completed. All data sharing will comply with privacy and confidentiality protections such as the NIH Certificate of Confidentiality and applicable laws, regulations, and policies governing data derived from human participants. Data requests should be addressed to the corresponding author at graboyes@musc.edu
